# Merocyanine and Spiropyran Adsorption on Graphene

**DOI:** 10.1021/acsomega.5c02292

**Published:** 2025-06-17

**Authors:** Olivia Bushman, Andreas Riemann

**Affiliations:** Department of Physics & Astronomy, 1632 Western Washington University, 516 High St, Bellingham, Washington 98225, United States

## Abstract

We have investigated the adsorption of spiropyran-based molecular switches on a graphene substrate using a combination of quantum-chemical and force-field-based calculations. Four different spiropyran molecules and their merocyanine isomers have been studied regarding the influence of different end groups on the surface adsorption and intermolecular configurations. Following the investigation of the adsorption behavior of single molecules, we expanded the study to the dimer formation of two identical molecules. This configuration is thought of as the precursor for substrate-wide film growth of the spiropyran-based molecular switch. The adsorption mechanisms and dimer formations can be understood due to an interplay of different parts of the molecules through van der Waals interactions and electrostatic forces from partial atomic charges of parts of the molecules. Furthermore, for each molecule, dimers based on different conformers of the merocyanine isomers could be identified.

## Graphical abstract

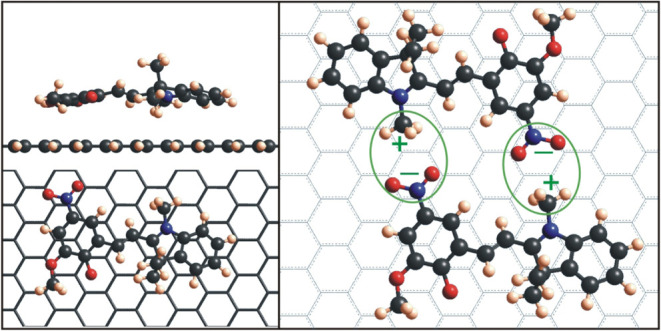

## 1 Introduction

The ultimate goal of the miniaturization of functional surface architectures is dependent on the choices for substrates and adsorbates.^1–6^ Many different classes of substrates can be employed: pure metals, conducting materials deposited on glass or plastics, semiconductors, insulators, insulating films deposited on conductors, etc.^7–13^ Among all of these options, graphene has generated an enormous interest over the last two decades.^14–18^ Its physical and electric properties have given rise to many research projects that use it as the substrate material.^19–22^ In this study, we add to the vast interest by employing graphene as a substrate material for spiropyran-based molecular switches. While graphene is a relatively new subject of research interest, spiropyran-based molecular switches have long been investigated, however, mostly in solution.^23–25^ Anchoring these molecules to a substrate can quench some of the switching mechanism and is therefore of profound interest when considering these molecules as a basis for molecular electronics.^26–28^

The current study uses the combination of quantum-chemical calculations with force-field approaches to computationally study the adsorption and dimer formation of spiropyran and merocyanine molecules on single-layer graphene substrates. Previous studies have shown that adsorption is driven by substrate–molecule interactions depending on the nature and configuration of the substrate and the adsorbate.^29–31^

To investigate the adsorption of molecules on the substrate with force-field calculations, substrates and molecules must be modeled in such a way that plausible charge configurations at both entities are present. For this approach, we have employed Density Functional Theory (DFT) for the molecules and the graphene substrate. In subsequent steps, we investigate the adsorption of single molecules and then the dimer formation of two adsorbed molecules with Molecular Mechanics (MM) calculations. These dimers were, in fact, experimentally observed and were the precursor to the substrate-wide film growth of the spiropyran-based molecular switches.

## 2 Methods

The investigations in this study use a combination of density functional theory (DFT) to determine partial atomic charges for the molecules and the substrate, together with molecular mechanics (MM) calculations to explore adsorption geometries and binding energies. When studying the adsorption of molecules on substrates, the size of the underlying substrate needs to be large enough to avoid so-called edge effects.^31,32^ In our case, a substrate of about 100 Å × 100 Å would be big enough to avoid any kind of influence from the edges. However, such a graphene substrate would have more than 4000 atoms, and the computational needs using DFT were not available. Therefore, we studied smaller substrates of graphene to determine the charge configuration of the graphene template and then extrapolated the findings about charges to a sufficiently large enough substrate. For this approach, we employed the Gaussian computational suite with the DFT-B3LYP method and 6–31G basis set.^33^ The self-consistent field (SCF) convergence was set to the default for Gaussian of SCF = Tight, leading to an energy convergence of 1.00D-06. The partial atomic charges were calculated using Mulliken charges and from the electrostatic potential (ESP) according to the ChelpG approach.^34^ With these methods, we investigated various sizes of graphene substrates.

All molecules and their conformers were optimized with the same parameter set as that of the substrates. Again, we employed the B3LYP method due to its widely recognized high quality for molecular chemistry calculations.^35^ The partial atomic charges on the molecules were determined with the ChelpG method as described in a previous study.^36^ The optimized geometries as minimum energy states were also verified through vibrational frequency analysis in Gaussian.

The molecules for this study were four spiropyran-based molecules and their merocyanine conformers in a *trans* configuration. These molecules can be seen in Figure 1: (a) benzo spiropyran and merocyanine (benzo SP & MC), from 1′,3′,3′-trimethylspiro­[1­(2*H*)-benzopyran-2,2′-indoline] (C_19_ H_19_ NO), (b) naphtho spiropyran and merocyanine (naphtho SP & MC), from 1,3,3-trimethylspiro­[indoline-2,3′-[3*H*] naphth [2,1-*b*] pyran] (C_23_ H_21_ NO), (c) nitro spiropyran and merocyanine (nitro SP & MC), from 1′,3′,3′-trimethyl-6-nitrospiro [1­(2*H*)-benzopyran-2,2′-indoline] (C_19_ H_18_ N_2_ O_3_), and (d) methoxy spiropyran and merocyanine (methoxy SP & MC), from 1′,3′-dihydro-8-methoxy-1′, 3′, 3′-trimethyl-6-nitrospiro [2*H*-1-benzopyran-2,2′-[2*H*]­indole] (C_20_ H_20_ N_2_ O_4_). In general, the merocyanine molecules can be found in eight different conformers, according to their three central carbon bonds being either in the *trans* or in the *cis* orientation. The naming scheme of the conformers is established according to these three bond configurations using the nitrogen in the pyrrole ring and the oxygen directly attached to the benzene ring as start and end points, respectively (Figure 2). The conformational flexibility of these merocyanine conformers separates them into the two aforementioned groups of T- and C-conformers. Their geometric properties lead to distinguished experimental outcomes when adsorbed onto a substrate. In the gas phase or solution, the T-conformers are very planar, whereas the C-conformers are helical/twisted in their structure with opposing end groups situated close to each other. As shown in an earlier spectroscopic study, this geometry leads to up to 1.65 kcal/mol higher adsorption energy for C-conformers compared to T-conformers.^37^ In an extensive, prior computational study by our group, we found the same behavior for C-conformers with consistently 0.2–0.3 eV higher adsorption energies compared to T-conformers.^36^ Therefore, when considering merocyanine molecular switches adsorbed on the graphene substrate, we decided to utilize only a subset of all conformers, namely T-conformers. In Figure 2, only these four conformers with the central bond in *trans* configuration are depicted. The choice of four different SP/MC molecules encompasses a wide range of side groups (naphtho rings, methoxy groups, and nitro groups) added onto the benzo SP/MC molecule to broaden the scope of the present investigation. These four compounds are also commercially available from TCI Chemicals.

**Figure 1:**
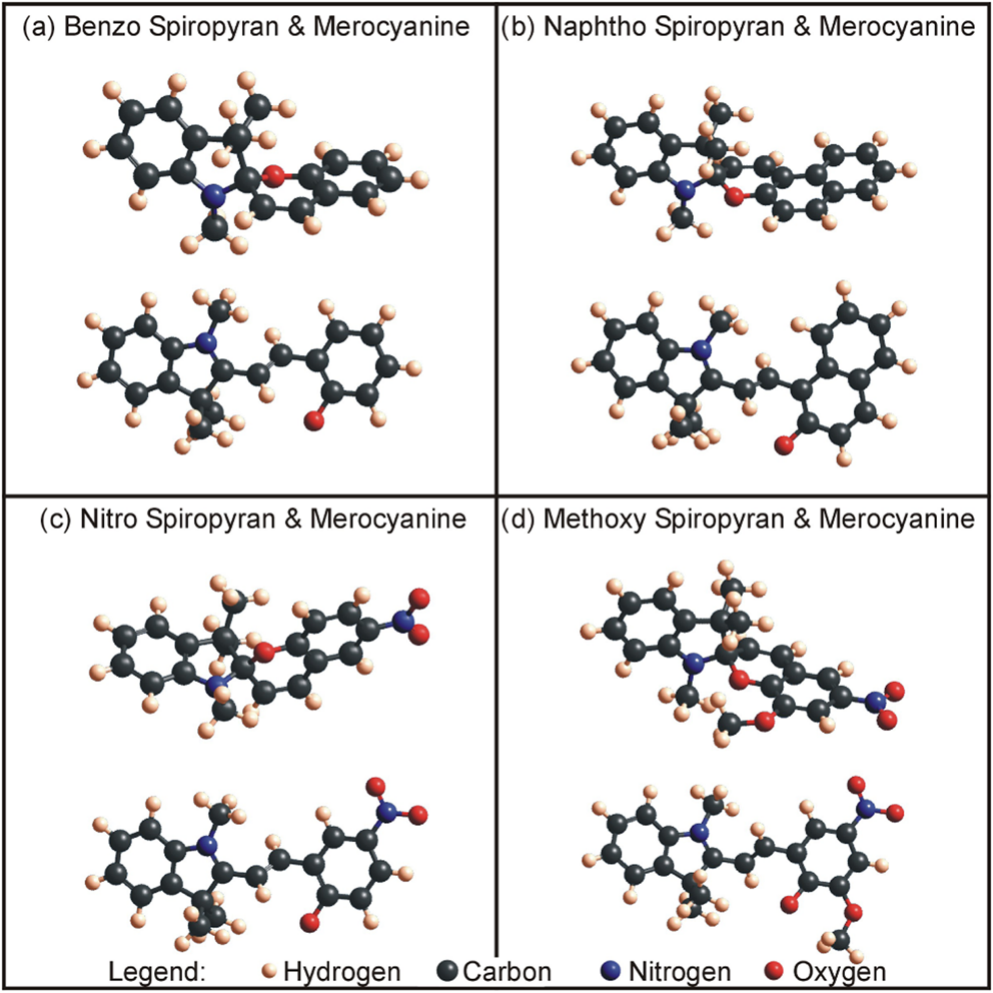
Four investigated molecules as spiropyran (SP) and respective merocyanine (MC) isomers: (a) benzo SP & MC from 1,3,3-trimethylindolinobenzopyrylospiran, (b) naphtho SP & MC from 1,3,3-trimethylindolino-β-naphthopyrylospiran, (c) nitro SP & MC from 1,3,3-trimethylindolino-6′-nitrobenzopyrylospiran, and (d) methoxy SP & MC from 1′,3′-dihydro-8-methoxy-1′,3′,3′-trimethyl-6-nitrospiro­[2*H*-1-benzopyran-2,2′-[2*H*]­indole]

**Figure 2:**
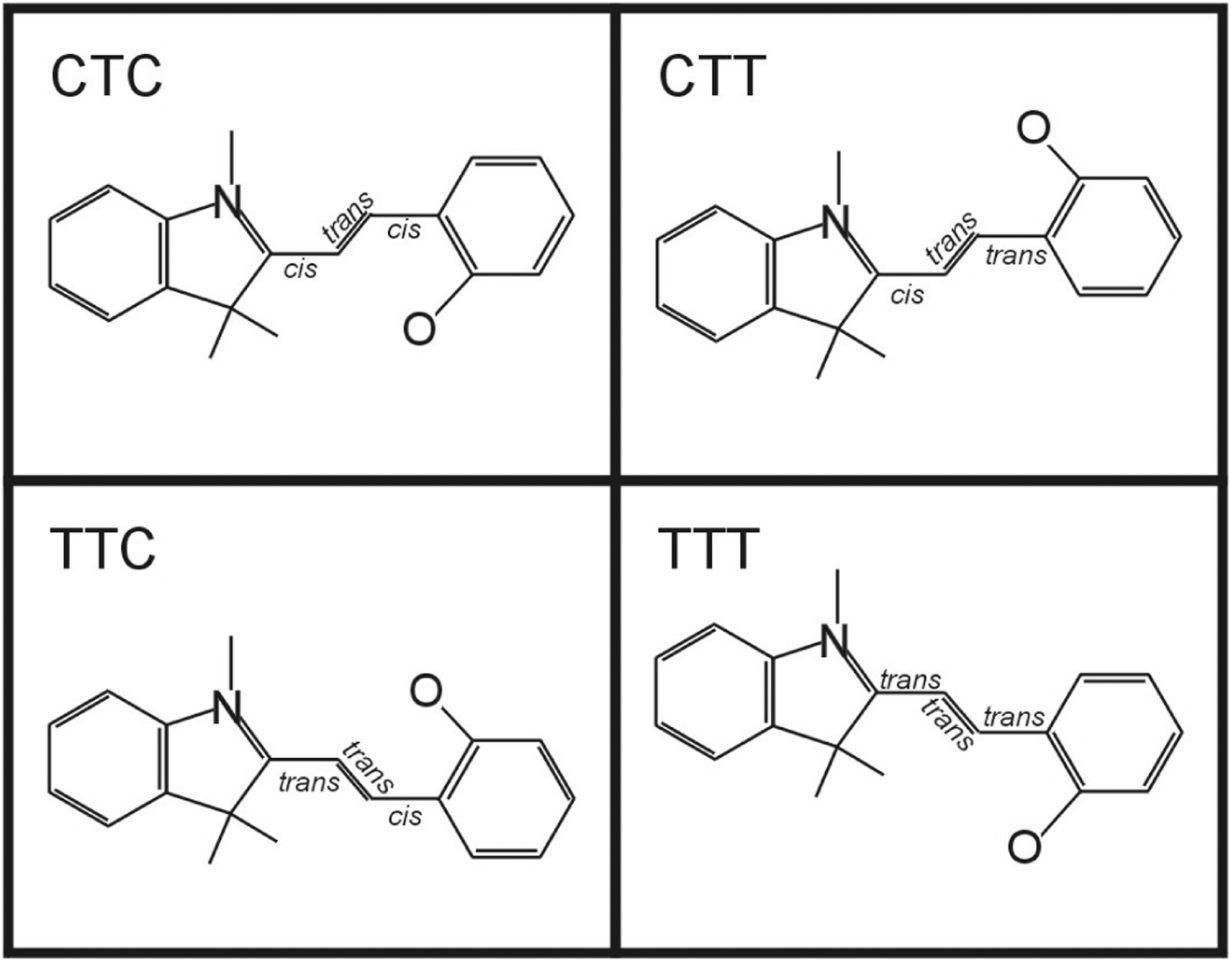
Naming scheme for merocyanine with central C–C bond in *trans* configuration (T-conformers, shown here as an example, benzo MC isomers). The labeling is according to the C–C bonds beginning with respect to nitrogen in the pyrrole ring and ending with respect to carbon attached to oxygen from the original pyran ring in SP.

After creating a sufficiently large graphene substrate with suitable partial atomic charges, the DFT-optimized molecules were the starting point of the calculations with molecular mechanics (MM) using HyperChem 8.0.^38^ As force fields for the MM calculation, AMBER 3 has been a reliable method to investigate adsorption energies and configurations as well as binding energies for multiple molecules.^29,39,40^ To find adsorption energies, the molecules were positioned close to the substrate (at a consistent height of 5 Å with their center of mass above the substrate) and allowed to find the optimal position using Polak–Ribiere energy-gradient calculation in HyperChem with a convergence limit of 0.001 kcal/mol.^38^ Adsorption energies were determined as the difference in energy for the substrate/adsorbate system between the situation where the molecule is very far away (200 Å above the graphene substrate) and when the molecule is in its optimal configuration on top of the substrate.

These optimally adsorbed molecules were then the starting points to determine the dimer configurations. From previous experimental results, we found that the beginning of film growth occurs with the formation of dimers, where larger clusters of more than two molecules were experimentally not observed.^41^ This indicates that these dimers need to consist of two molecules that are in antiparallel configuration, meaning that the two molecules are rotated by 180° with respect to the surface normal. In this current study, we accomplished this situation by fixing one molecule (its center of mass being the origin of our established coordinate system) and then moving a second, identical, but rotated molecule around the origin on a grid of 20 Å × 20 Å in 0.1 Å step size in both lateral directions and recording the energy for each position. The optimal configuration is at the location of the lowest energy. The system’s energy with the two molecules situated at the aforementioned positions is compared to the system’s energy with both molecules adsorbed on the surface at equivalent lattice positions but located so far away from each other that they do not interact. The difference is considered the binding energy for this antiparallel dimer.

## 3 Results and Discussion

### 3.1 Graphene Substrate

In order to investigate the adsorption of spiropyran-based molecules on graphene, a valid substrate configuration with appropriate partial atomic charges is needed for the molecular mechanics calculations. Besides the requirements for partial atomic charges, the substrate also needs to be large enough so that edge effects can be avoided; previous studies have shown that a lateral size of 100 Å × 100 Å would be sufficient.^31^ However, such a substrate has >4000 atoms and would not be workable for DFT calculations. Therefore, our first step was to create a suitable substrate through systematic extrapolation from smaller substrates. We investigated graphene substrates with 54 carbon atoms (about 10 Å × 10 Å), 170 carbon atoms (about 20 Å × 20 Å), and 432 carbon atoms (about 30 Å × 30 Å), all terminated with hydrogens at the edges to achieve valence saturation. Analyzing the vibrational frequencies obtained in Gaussian verifies that the calculations of the substrate’s structure produced the minimum energy configuration. We used DFT-B3LYP and a 6–31G basis set. Charges were determined according to the well-established Mulliken and ChelpG ESP methods. The result for the largest substrate investigation of the individual charges is shown in Figure 3. Inside carbons have calculated charges very close to zero, and outside carbons have a slightly negative charge, with the terminating hydrogen atoms carrying a slightly positive charge. Using the results of the largest possible configuration for which we were able to carry out DFT calculations, we created a template with the following parameters: hydrogen charges are +0.136*e* (the average determined from the largest optimized substrate), the connected carbons have the opposite charge of −0.136*e*, the carbon–hydrogen distance is 1.084 Å, and the carbon–carbon distances are 1.431 Å. All of these values are consistent with previous findings.^42,43^ As mentioned before, the calculated charges for the inner carbons fluctuated around zero charge. In order to investigate this effect, we created four different substrates with inner charges of ± 0*e*, ± 0.005*e*, ± 0.010*e*, and ± 0.020*e*; neighboring carbons were given opposite charges in order to keep the graphene sheet overall electrically neutral. With these parameters, we constructed four large enough substrates as starting points for our adsorption studies. This substrate consists of 3984 carbon atoms and 178 hydrogen atoms, terminating the graphene sheet on the outside. The dimensions are about 100 Å × 100 Å.

**Figure 3:**
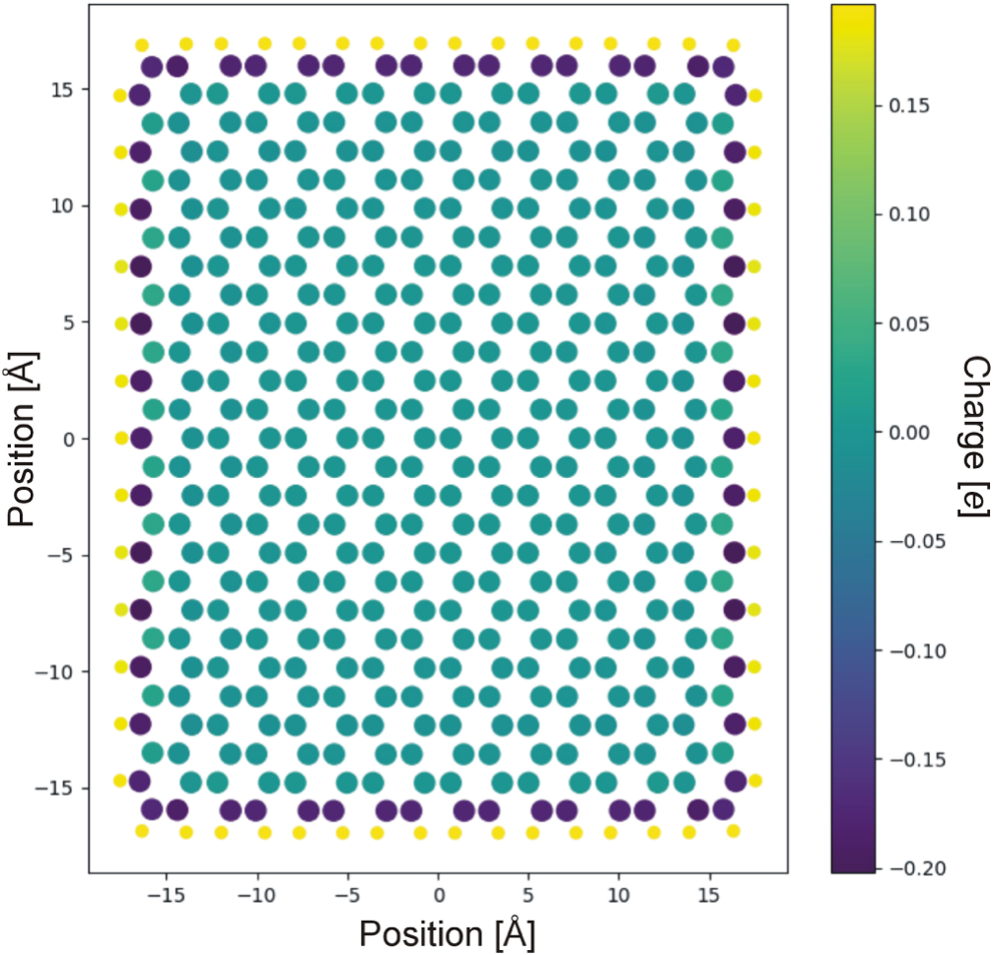
Map of graphene substrate after optimization and partial atomic charge determination using DFT-B3LYP and a 6–31G basis set, and 432 carbon atoms are used. The inner carbon atoms have acquired only a minimal charge (less than ± 0.02*e*). Hydrogen atoms and connected outer carbon atoms acquired complementary (opposite charges).

### 3.2 Adsorption

Using the benzo merocyanine molecules and the four substrates with slightly different charges on the inner carbons (±0*e*, ± 0.005*e*, ± 0.010*e*, and ± 0.020*e*), we investigated the influence of the adsorption energy and geometry. We found no substantial differences in adsorption geometry when using the four substrates with different charges on the inner carbons, and the adsorption energies only varied by less than 0.01 eV. For the remainder of the study, we focused on the substrate with a charge of ± 0.01*e* on the inner carbon atoms. The molecules were optimized with DFT-B3LYP and using the ChelpG ESP method for their charge, and the molecular mechanics calculations were carried out with the AMBER 3 force field.

In general, the spiropyran with its spiro intersection and two perpendicular rings is a three-dimensional molecule, whereas the T-conformer of the merocyanine isomer is two-dimensional with a nearly flat geometry. This can also be observed in their adsorption behavior (see Figure 4). There, one can clearly see (with the example of the methoxy SP and TTT merocyanine isomers) that only some of the aromatic rings of spiropyran participate in the interactions with the surface. On the other hand, the merocyanine molecule lies nearly flat on the surface, and all three aromatic rings can contribute to the adsorption, which is manifested in the adsorption energies (see Table 1). Previous studies have shown that the number of aromatic rings involved in these binding processes is strongly related to adsorption energies.^44^ For the benzo molecules, SP has about 2/3 of the adsorption energy of MC, reflecting the fact that benzo SP has two aromatic rings parallel to the surface, but benzo MC has three rings contributing to the energy. A similar situation can be observed for the naphtho molecules: here, SP has three aromatic rings nearly parallel to the substrate, but MC has four rings contributing to the binding. For the nitro and methoxy molecules, besides the aromatic rings, the methoxy and nitro groups also contribute to the energy. Comparing the number of aromatic rings in SP for binding and in MC for binding, the ratio is the same as that for the benzo molecule, two to three. Nevertheless, methoxy SP shows overall a higher binding energy due to the contributions of the additional methoxy group. From the molecular data, we can get rough estimates of binding energy contributions for different constituents of the molecules: an aromatic ring (isolated extra contributions when comparing naphtho with benzo) adds about 0.2–0.3 eV to the binding to graphene, the nitro group about 0.1 eV, and the methoxy group another 0.2 eV. We can compare these values to other studies and see that, depending on the actual adsorption position (bridge, hollow, stack), energies between 0.24 and 0.30 eV have been found for these aromatic rings in agreement with our findings.^45,46^ For NO_2_ studies on graphene, a relatively low adsorption energy of 0.06 to 0.08 eV has been reported, consistent with our rough estimate of about 0.1 eV.^47,48^ To compare the contribution of the methoxy group to the adsorption energy, one could look at methanol adsorption, and note that energies of about 0.2 eV were found, again comparable to the values estimated by comparing the different SP and MC molecular adsorption energies.^49^

**Table 1:** Adsorption Energies (in eV) for All Four Molecules and the Spiropyran and Merocyanine Isomers with Various Conformers

molecule	SP	CTC	CTT	TTC	TTT
benzo	1.06	1.49	1.49	1.48	1.57
naphtho	1.32	1.87	1.86	1.57	1.70
nitro	1.15	1.74	1.64	1.65	1.67
methoxy	1.36	1.87	1.86	1.86	1.81

**Figure 4:**
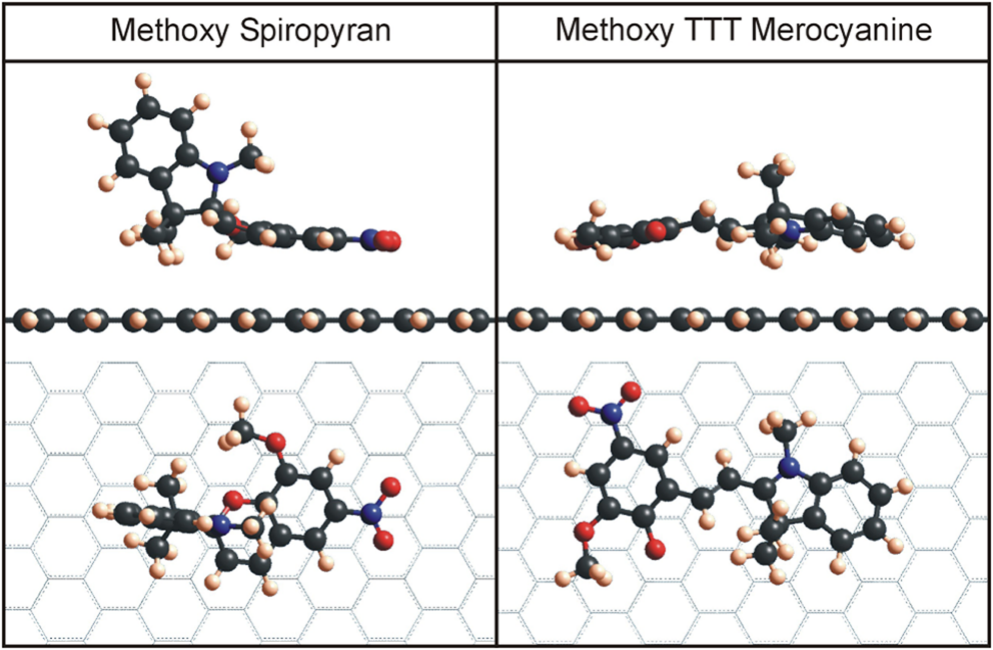
Top view and side view of representative geometries of spiropyran molecules and merocyanine conformers (pictured here are methoxy spiropyran and methoxy merocyanine as TTT conformers). The spiropyran has two aromatic rings parallel to the underlying graphene substrate and two rings perpendicular to it, resulting in overall lower adsorption energy than, for instance, the TTT conformer of methoxy merocyanine, where all three aromatic rings are nearly parallel to the graphene substrate.

For all four molecules, the SP molecules have consistently lower adsorption energies than the MC molecules. Extra end groups in the MC molecules contribute additionally to the adsorption energies. Overall, a flat adsorption geometry of merocyanine on graphene has been observed, whereas SP’s adsorption geometry is more varied.

### 3.3 Dimer Formation

Previous experimental results of naphtho MC adsorption on Au(111) investigated with Scanning Tunneling Microscopy, showed that merocyanine dimers are formed upon adsorption on the surface.^41^ Remarkably, no larger clusters than these of two molecules can be initially observed, which indicates that these two molecules forming a dimer should be in antiparallel orientation, meaning one is rotated by 180° with respect to the surface normal. Taking these observed configurations as a starting point to investigate which conformers might be energetically most favorable to create these dimers, we studied the dimer formation with two molecules in an antiparallel configuration. As described above, we used two optimally adsorbed, identical molecules and moved the second (rotated) molecule on a grid to find the lowest energy position. The results for the four spiropyran molecules can be seen in Figure 5, and results for the four T-conformers of all four molecules can be seen in Figures 6–9. An overview of the binding energies, calculated from the difference between the system’s energy with two molecules adsorbed on the surface but not interacting and the system’s energy when these molecules are optimally positioned, can be seen in Table 2.

**Figure 5:**
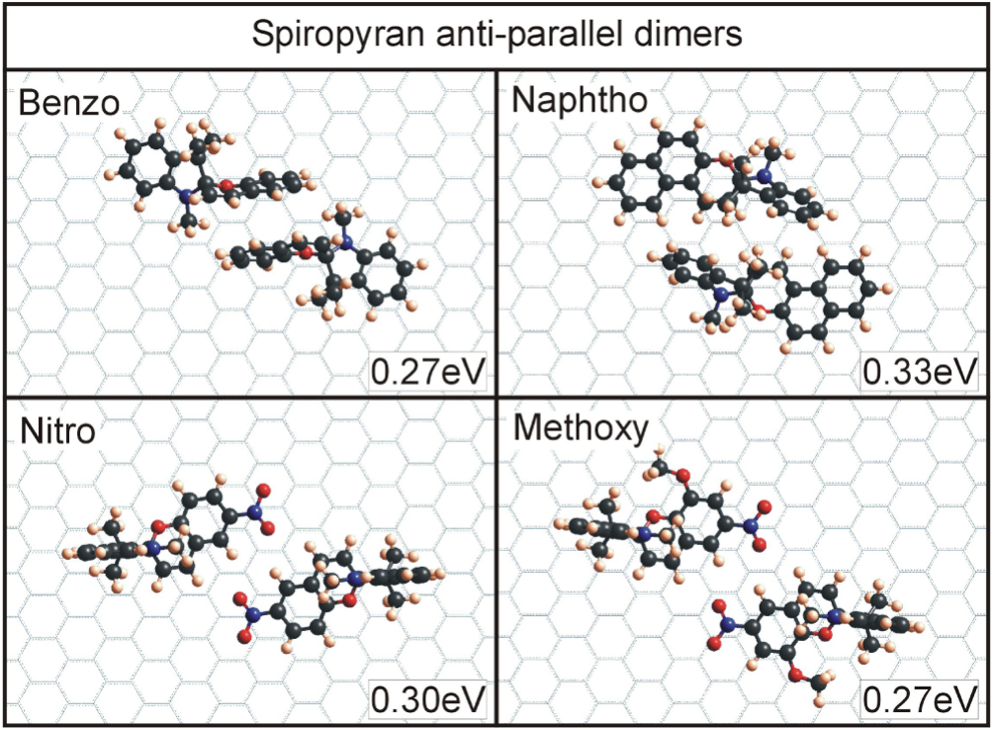
Dimers for the spiropyran molecules. The benzo SP molecules arrange in such a way that the aromatic rings, which are perpendicular to the surface, are close to each other. For the other three dimer configurations, a clear reasoning for their preferred geometry can not be deduced.

**Figure 6:**
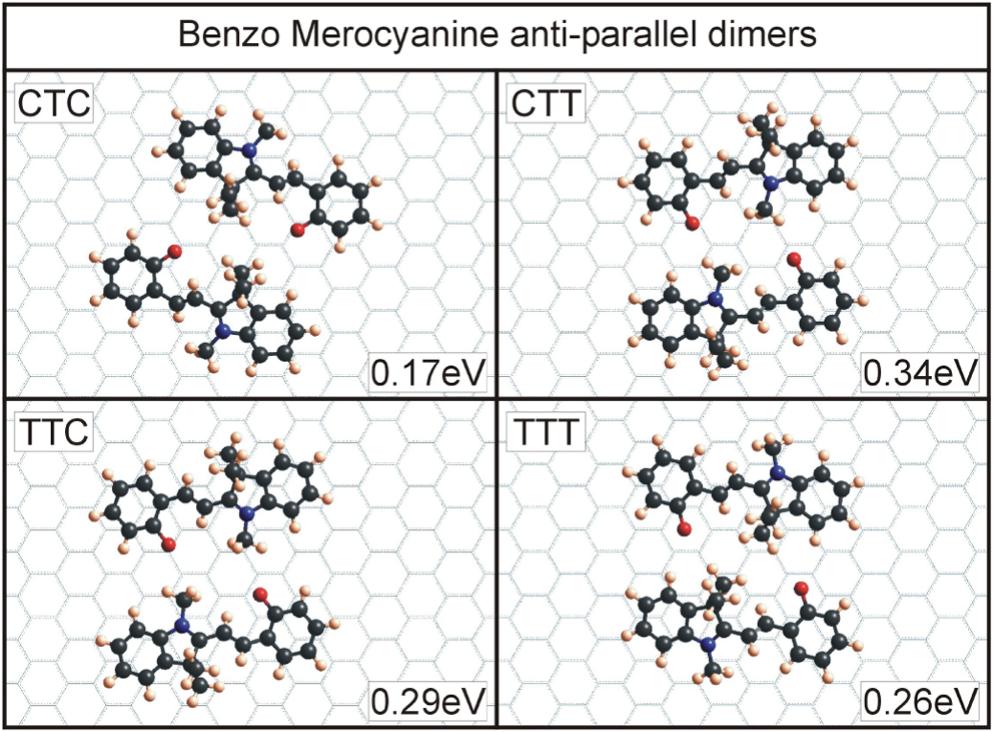
Dimers for benzo merocyanine with the four T-conformers. One can see the higher binding energies for CTT and TTC dimers due to their geometry of having electrostatic interactions between the oppositely charged O-21 and methyl C-12 entities.

**Table 2:** Binding Energies (in eV) for All Four Molecules and Their Dimer Configurations in Antiparallel Orientation

molecule	SP	CTC	CTT	TTC	TTT
benzo	0.27	0.17	0.34	0.29	0.26
naphtho	0.33	0.16	0.30	0.25	0.22
nitro	0.30	0.67	0.47	0.38	0.58
methoxy	0.27	0.69	0.30	0.30	0.58

In order to understand these different geometries, it is helpful to look at the partial atomic charges for each molecule. The following trend has been documented in a previous study.^36^ For the merocyanine isomers: the oxygen atom (labeled O-21 in the previous study), which was part of the SP pyran ring, carries a negative charge, the methyl group (C-12) attached to the nitrogen in the pyrrole ring carries an overall positive charge, and the other oxygens in the nitro functional group, part of the nitro SP/MC and methoxy SP/MC molecules, also carry a negative charge. More details on these charge distributions can be found in the Supporting Information to our previous study.^36^ These partial atomic charges play an important role through Coulomb interactions to determine the energetically most favorable situations when two molecules interact as dimers.

Looking at the dimer formation for all four spiropyran isomers in Figure 5, one can not decipher a common configuration. For benzo SP, the rings that are perpendicular to the substrate interact in the dimers with each other and contribute significantly to the binding energy. For the other three SP dimers, a similar binding energy can be found; however, the exact nature of the interaction is hard to pinpoint. Overall, the binding energies are fairly consistent with about 0.30 ± 0.03 eV for all four molecules.

Looking at the dimers for benzo MC (Figure 6), the dimers for CTT and TTC are the two with the highest binding energies. Here, the aforementioned partial charges on the O-21 atom and the C-12 methyl group are close together and provide Coulomb interactions to increase binding energy. The geometries of the other two dimers do not allow for this configuration, and therefore, other interactions provide a lower binding energy. If, as experimentally observed for a slightly different system, only two different MC dimers are present, the energetically most favorable would be CTT and TTC dimers for the benzo MC.

Similar findings can be observed for naphtho MC dimers; see Figure 7. Also, here, the CTT and TTC dimers have the highest binding energy, and the negatively charged O-21 atom is again close to the positively charged C-12 methyl group. The additional benzene ring, which distinguishes the naphtho SP/MC from the benzo SP/MC, does not influence the dimer formation significantly. Although the other two dimers, consisting of CTC and TTT conformers, have lower overall binding energies, it can be observed here, as well as for benzo MC, that the oxygen atom O-21 is always on the inner side of the dimer formation, indicating an important role in facilitating the interactions between two merocyanine molecules.

**Figure 7:**
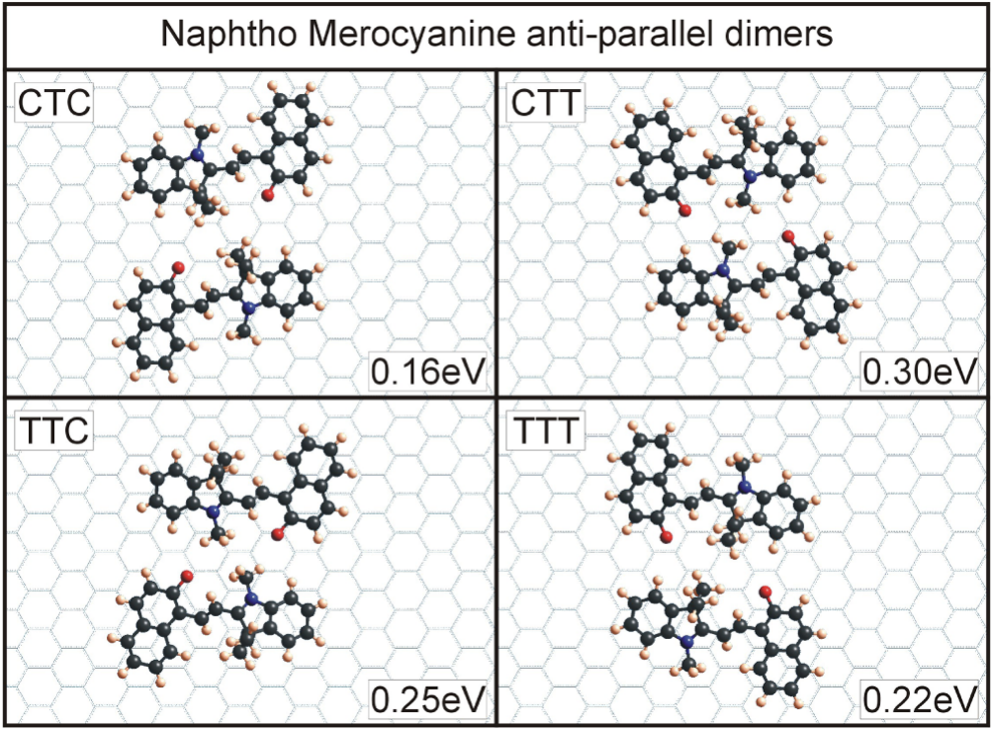
Dimers for naphtho merocyanine with the four T-conformers. Similar to the benzo MC, CTT and TTC are energetically most favorable with oppositely charged parts of the molecules (O-21 as negative and the C-12 methyl group as positive) in proximity. This configuration leads to an added Coulomb interaction during the dimer formation. The CTC and TTT dimers are not conducive to this arrangement and have, therefore, smaller binding energies. Notice, though, that for all four dimers, the negatively charged O-21 moiety is on the inside of the arrangement, contributing to the overall binding.

For nitro MC (Figure 8), one can see that the dimers consisting of CTC and TTT conformers have the highest binding energies. In these cases, the attractive interactions between the two molecules are facilitated by the negatively charged oxygens in the nitro functional group and the positive methyl group. Since these interactions contribute more to the binding than the Coulomb interactions between the oxygen, O-12, and the methyl group, the two conformers CTC and TTT produce energetically more favorable dimers in the case of nitro MC. Nevertheless, the other two conformers create dimers with similar binding energies as observed for the other molecules, benzo and naphtho MC. Again, the overarching driving force for the dimer formation for these molecules is Coulomb interactions between oppositely charged parts of the molecule.

**Figure 8:**
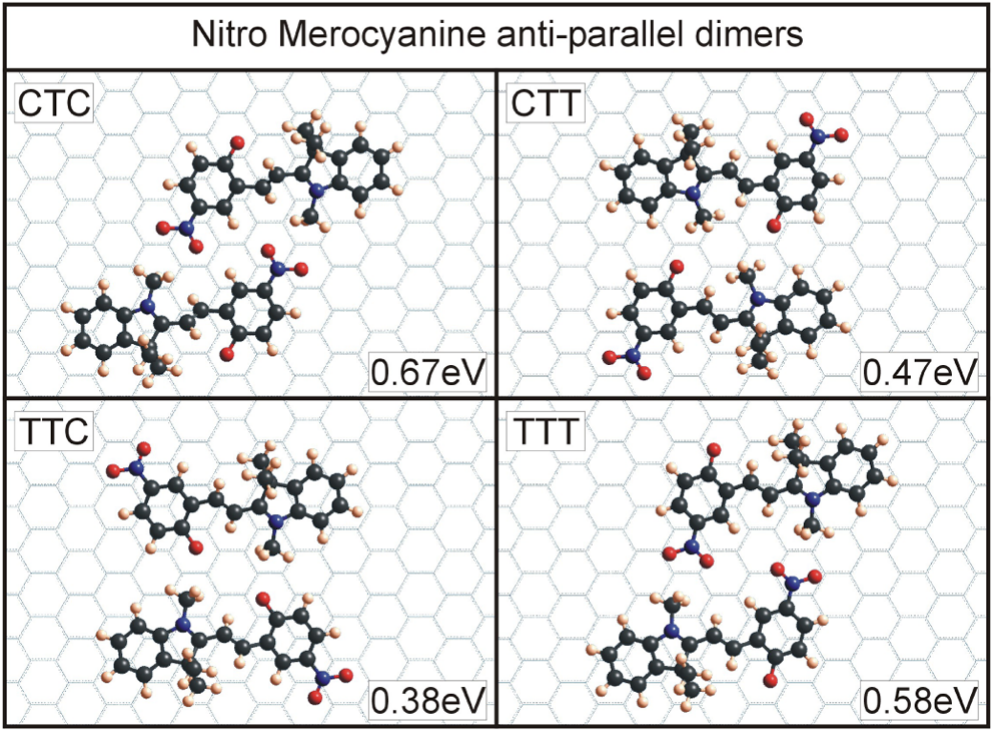
Dimers for nitro merocyanine with the four T-conformers. In this case, the CTC and TTT dimers show the highest binding energies. Here, the oppositely charged parts of the molecule, the methyl group C-12 and the oxygens in the nitro group, are positioned in such a way as to contribute to the binding energy via Coulomb interactions.

The last merocyanine dimers investigated consisted of the methoxy MC conformers, as seen in Figure 9. Similar to nitro MC, the CTC and TTT dimers are energetically most favorable. The geometry of these dimers allows the positively charged oxygens in the nitro groups and the negatively charged methyl groups to be close enough to substantially contribute to the binding energies via Coulomb interactions. The other two dimers, consisting of CTT and TTC conformers, only have about half of the binding energies since none of the aforementioned charged parts of the molecules are able to be positioned close enough to contribute more strongly to the binding.

**Figure 9:**
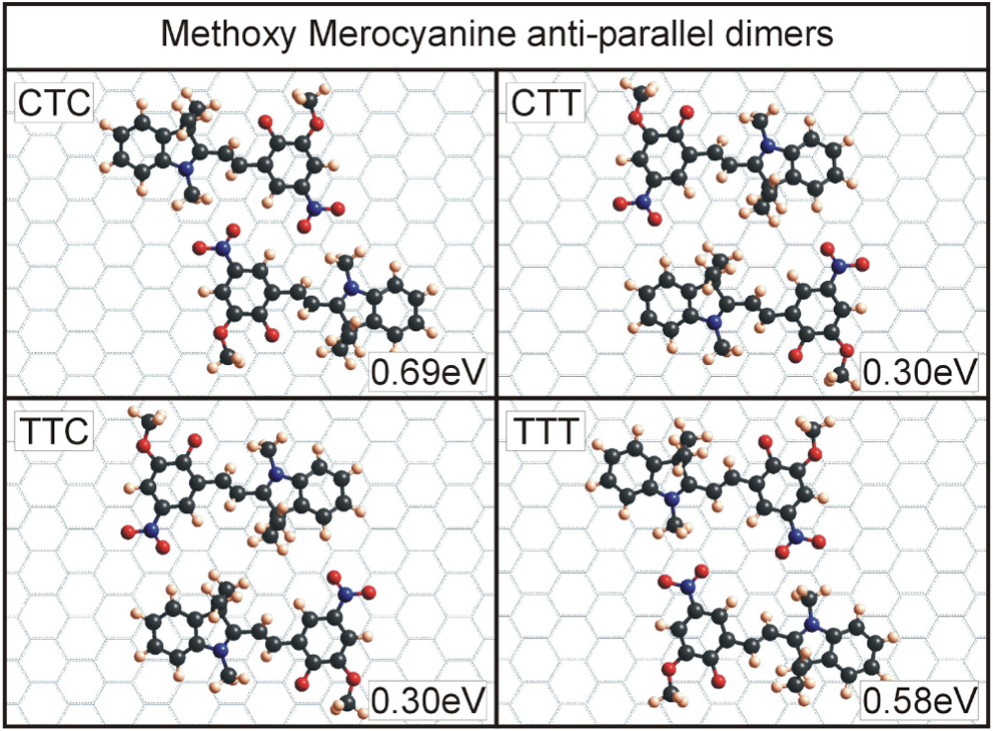
Dimers for methoxy merocyanine with the four T-conformers. Similar to the nitro MC, CTC and TTT dimers are the energetically most favorable due to Coulomb interactions of the methyl group C-12 and the oxygens in the nitro group.

For all four molecules, two merocyanine dimers could be identified, which are energetically more favorable to be created when adsorbed on a graphene substrate. The driving forces are Coulomb interactions of oppositely charged parts of the molecules. The geometry of the individual SP/MC molecule plays a role, as for the very similar benzo and naphtho MCs, CTT and TTC conformers are more likely, and for the similar nitro and methoxy MCs, CTC and TTT are more likely.

## 4 Conclusions

In this study, we have investigated four different spiropyran-based molecules and their adsorption on a graphene substrate. After creating a suitable graphene template, we found adsorption geometries and energies that are consistent with the original configurations: spiropyran molecules adsorb with part of their aromatic rings parallel to the substrate and other rings perpendicular to it, therefore having an overall lower adsorption energy than their merocyanine counterparts. These merocyanine molecules adsorb nearly flat or parallel to the substrate, and therefore, more moieties of the molecule partake in the adsorption process, leading to overall higher adsorption energies.

When looking at the dimer formation of two identical molecules that can be thought of as the precursor to subsequent film growth, our study shows that for each of the four different SP/MC molecules, two conformers of merocyanine could be identified as energetically most favorable due to intermolecular interactions partly driven by Coulomb interactions of oppositely charged parts of the molecules. These findings are consistent with previous experimental results of naphtho merocyanine on an Au(111) substrate.

## References

[1] Barth J. V., Weckesser J., Cai C., Günter P., Bürgi L., Jeandupeux O., Kern K. (2000). Building Supramolecular
Nanostructures at Surfaces by Hydrogen Bonding. Angew. Chem., Int. Ed..

[2] Enders A., Skomski R., Honolka J. (2010). Magnetic surface
nanostructures. J. Phys.:Condens. Matter.

[3] Barth J. V., Constantini G., Kern K. (2005). Engineering atomic and molecular
nanostructures at surfaces. Nature.

[4] Knez M., Nielsch K., Niinistö L. (2007). Synthesis
and Surface Engineering
of Complex Nanostructures by Atomic Layer Deposition. Adv. Mater..

[5] Gomar-Nadal E., Puigmartí-Luis J., Amabilino D. B. (2008). Assembly
of functional molecular nanostructures on surfaces. Chem. Soc. Rev..

[6] Barth J. V. (2007). Molecular
Architectonic on Metal Surfaces. Annu. Rev.
Phys. Chem..

[7] Rosei F., Schunack M., Naitoh Y., Jiang P., Gourdon A., Laegsgaard E., Stensgaard I., Joachim C., Besenbacher F. (2003). Properties
of large organic molecules on metal surfaces. Prog. Surf. Sci..

[8] Tang H., Shen Z., Shen Y., Yan G., Wang Y., Han Q., Han L. (2024). Reinforcing self-assembly
of hole transport molecules
for stable inverted perovskite solar cells. Science.

[9] Wang L., Schubert U. S., Hoeppener S. (2021). Surface chemical reactions on self-assembled
silane based monolayers. Chem. Soc. Rev..

[10] Fölsch S., Helms A., Riemann A., Repp J., Meyer G., Rieder K. H. (2002). Nanoscale surface
patterning by adsorbate-induced faceting
and selective growth: NaCl on Cu(211). Surf.
Sci..

[11] Auwärter W., Weber-Bargioni A., Riemann A., Schiffrin A., Gröning O., Fasel R., Barth J. V. (2006). Self-assembly and
conformation of tetrapyridyl-porphyrin molecules on Ag(111). J. Phys. Chem. A.

[12] Cho S. W., Piper L. F. J., DeMasi A., Preston A. R. H., Smith K. E., Chauhan K. V., Sullivan P., Hatton R. A., Jones T. S. (2010). Electronic
Structure of C_60_ /Phthalocyanine/ITO Interfaces Studied
using Soft X-ray Spectroscopies. J. Phys. Chem.
C.

[13] Riemann A., Fölsch S., Rieder K. H. (2005). Epitaxial growth of alkali halides
on stepped metal surfaces. Phys. Rev. B.

[14] Gao L., Guest J. R., Guisinger N. P. (2010). Epitaxial
Graphene on Cu(111). Nano Lett..

[15] Günther S., Dänhardt S., Wang B., Bocquet M.-L., Smitt S., Wintterlin J. (2011). Single Terrace
Growth of Graphene on a Metal Surface. Nano
Lett..

[16] Batzill M. (2012). The surface
science of graphene: Metal interfaces, CVD synthesis, nanoribbons,
chemical modifications, and defects. Surf. Sci.
Rep..

[17] Bode N., Mariani E., von Oppen F. (2012). Transport properties of graphene
functionalized with molecular switches. J. Phys.:
Condens. Matter.

[18] Novoselov K. S., Fal’ko V. I., Colombo L., Gellert P. R., Schwab M. G., Kim K. (2012). A roadmap
for graphene. Nature.

[19] Zan R., Bangert U., Ramasse Q., Novoselov K. S. (2011). Evolution
of Gold Nanotsructures on Graphene. Small.

[20] Deshpande A., Sham C., Alaboson J. M. P., Mullin J. M., Schatz G. C., Hersam M. C. (2012). Self-Assembly and
Photopolymerization of Sub-2 nm One-Dimensional
Organic Nanostructures on Graphene. J. Am. Chem.
Soc..

[21] Järvinen P., Hamäläinen S. K., Banerjee K., Häkkinen P., Ijäs M., Harju A., Liljeroth P. (2013). Molecular
Self-Assembly on Graphene on SiO_2_ and h-BN Substrates. Nano Lett..

[22] Jung M., Shin D., Sohn S.-M., Kwon S.-Y., Park N., Shin H.-J. (2014). Atomically resolved orientational ordering of C_60_ molecules on epitaxial graphene on Cu(111). Nanoscale.

[23] Fischer E., Hishberg Y. (1952). Formation of coloured forms of spirans by low-temperature
irradiation. J. Chem. Soc..

[24] Chaudé O., Rumpf P. (1953). The double phototropy of certain spiropyrans. C.R. Acad. Sci..

[25] Berkovic G., Krongauz V., Weiss V. (2000). Spiropyrans and Spirooxazines for
Memories and Switches. Chem. Rev..

[26] Piantek M., Schulze G., Koch M., Franke K. J., Leyssner F., Krüger A., Navío C., Miguel J., Bernien M., Wolf M., Kuch W., Tegeder P., Pascual J. I. (2009). Reversing
the Thermal Stability of a Molecular Switch on a Gold Surface: Ring-opening
reaction of Nitrospiropyran. J. Am. Chem. Soc..

[27] Bronner C., Schulze G., Franke K. J., Pascual J. I., Tegeder P. (2011). Switching
ability of nitro-spiropyran on Au(111): electronic structure changes
as a sensitive probe during a ring-opening reaction. J. Phys.: Condens. Matter.

[28] Schulze G., Franke K. J., Pascual J. I. (2012). Induction
of a Photostationary Ring-Opening-Ring-Closing
State of Spiropyran Monolayers on the Semimetallic Bi(110) Surface. Phys. Rev. Lett..

[29] Riemann A., Nelson B. (2009). Molecular wires self-assembled
on a graphite surface. Langmuir.

[30] Krebs E., Grabill L., Riemann A. (2018). Amino acid
nanopatterning on graphite. Surf. Sci..

[31] Thorpe J., Riemann A. (2022). Combined DFT and Molecular
Mechanics Modeling of the
Adsorption of Semiconducting Molecules on an Ionic Substrate: PTCDA
and CuPc on NaCl. ACS Omega.

[32] Grabill L., Riemann A. (2018). Conformational Impact
on Amino Acid-Surface *π*-*π* Interactions on a (7,7)
Single-Walled Carbon Nanotube: A Molecular Mechanics Approach. J. Phys. Chem. A.

[33] Frisch, M. J. ; Trucks, G. W. ; Schlegel, H. B. ; Scuseria, G. E. ; Robb, M. A. Gaussian; Gaussian, Inc.: Wallingford CT, USA, 2016.

[34] Breneman C. M., Wiberg K. B. (1990). Determining atom-centered
monopoles from molecular
electrostatic potentials - the need for high sampling density in formamide
conformational-analysis. J. Comput. Chem..

[35] Yu G., Lyu L., Zhang F., Yan D., Cao W., Hu C. (2018). Theoretical
and experimental evidence for rGO-4-PP Nc as a metal-free Fenton-like
catalyst by tuning the electron distribution. RSC Adv..

[36] Riemann A., Rankin L., Henry D. (2024). Atomic Charge
Dependency of Spiropyran/Merocyanine
Adsorption as a Precursor to Surface Isomerization Reactions. ACS Omega.

[37] Morley J. O., Morley R. M., Fitton A. L. (1998). Spectroscopic Studies
on Brooker’s
Merocyanine. J. Am. Chem. Soc..

[38] Hypercube, Inc. . 1115 NW 4th Street, 2011 Gainesville, Florida 32601, USA.

[39] Dasetty S., Barrows J. K., Sarupria S. (2019). Adsorption of Amino Acids on Graphene:
Assessment of Current Force Fields. Soft Matter.

[40] Dubbeldam D., Walton K. S., Vlugt T. J. H., Calero S. (2019). Design, Parameterization,
and Implementation of Atomic Force Fields for Adsorption in Nanoporous
Materials. Adv. Theory Simul..

[41] Riemann A., Browning L., Goff H. (2021). Conformational behavior of naphtho-merocyanine
dimers on Au(111). Surf. Sci..

[42] Abergel D., Apalkov V., Berashevich J., Ziegler K., Chakraborty T. (2010). Properties
of graphene: a theoretical perspective. Adv.
Phys..

[43] Wang J., Ma F., Sun M. (2017). Graphene, hexagonal boron nitride, and their heterostructures:
properties and applications. RSC Adv..

[44] Moriggi F., Barbera V., Galimberti M., Raffaini G. (2023). Adsorption Affinities
of Small Volatile Organic Molecules on Graphene Surfaces for Novel
Nanofiller Design: A DFT Study. Molecules.

[45] AlZahrani A. Z. (2010). First-principles
study on the structural and electronic properties of graphene upon
benzene and naphthalene adsorption. Appl. Surf.
Sci..

[46] Zhang Y.-H., Zhou K., Xie K., Zeng J., Zhang H., Peng Y. (2010). Tuning the electronic structure and transport properties of graphene
by noncovalent functionalization: effects of organic donor, acceptor
and metal atoms. Nanotechnology.

[47] Leenaerts O., Partoens B., Peeters F. M. (2008). Adsorption of H_2_O, NH_3_, CO, NO_2_, and NO on graphene:
A first-principles
study. Phys. Rev. B.

[48] Guo L., Hao Y., Li P., Song J., Yang R., Fu X., Xie S., Zhao J., Zhang Y. (2018). Improved NO_2_ Gas Sensing
Properties of Graphene Oxide Reduced by Two-beam-laser Interference. Sci. Rep..

[49] Schröder E. (2013). Methanol Adsorption
on Graphene. J. Nanomater..

